# Living with technological challenges: does socioeconomic status affect people’s health?

**DOI:** 10.1186/s12877-024-05662-2

**Published:** 2025-03-03

**Authors:** Jia Xu, Chun Xia, Xiuzhen Ding

**Affiliations:** 1 School of Marxism, Anhui Normal University, Jiuhua-Nan-Road 189, Wuhu, 241000 China; 2 School of Educational Science, Anhui Normal University, Jiuhua-Nan-Road 189, Wuhu, 241000 China; 3 School of History, Anhui Normal University, Jiuhua-Nan-Road 189, Wuhu, 241000 China

**Keywords:** Technological challenges, Socioeconomic status, Health condition, Medical care system, Rural–urban, China

## Abstract

**Background:**

Technological challenges in accessing medical care services may cause individuals to feel isolated from the medical care system. This study posits that individual’s subjective socioeconomic status (SES) contribute to differing levels of technological challenges when seeking medical care services, subsequently impacting their health conditions.

**Methods:**

A questionnaire survey was administered to 1,932 residents in China (1,037 men, 891 women, and 4 missing; *M*_age_: 64.28 ± 11.30 years, range: 45–99 years). Participants included 792 urban (40.99%) and 1,140 rural (59.01%) residents. We measured SES, technological challenges perceptions, health conditions, and other control variables.

**Results:**

Analysis of 10,000 bootstrapped samples revealed that technological challenges partially mediate the association between SES and health conditions. Moreover, rural people with low SES had poorer health because of technological challenges. This effect was not significant for urban people after controlling for sex, age, education level, marital status, and experience accessing medical care services.

**Conclusions:**

SES significantly and negatively impacted individuals’ health conditions, especially for rural residents, owing to their technological challenges. This study provides evidence and insights into the nexus of policy formulation, modern technology, and public perceptions regarding shortcomings and risks in public health policies.

## Background

In recent years, the implementation of modern technology in medical care services, such as online appointment booking and digital access to medical test results, has become popular [1–5], helping to improve service delivery efficiency and accelerated medical service utilization [6–8]. For example, patients can now access services, book appointments, or check test results more conveniently and quickly, instead of waiting long hours in medical care institutions [9–11]. However, scholars have argued that innovations may introduce access-related challenges for older adults or those with low educational levels [12–14], potentially excluding those who struggle with technology from effectively using medical care services [15–18]. 

Consequently, people’s health conditions may worsen because of their inability to access medical care services, and they may feel isolated owing to these challenges—especially those with a low socioeconomic status (SES) [19, 20]. SES is among the most important indicators for analyzing residents’ social policy utilization, studied extensively in fields like sociology, management, and other disciplines [21–24]. Therefore, it is essential to explore whether individuals with different SES perceive technological challenges differently when accessing medical care services [25]. 

Consequently, we are interested in how differences in urban–rural residency impact perceptions of technological challenges when accessing medical care resources across different SES levels. Scientific evidence highlights disparities in urban–rural medical resources within China, with residents highlights varying degrees of access to these resources, often influenced by regulatory design. This may partly owing to the unique technological challenges faced by rural residents in accessing medical resources [26], or owing to the association between SES and residents’ health conditions [20]. Considering this, we believe that maybe individuals with lower SES and greater technological challenges may face barriers to obtaining medical care information, seeking support, and using medical services [27]. However, despite an extensive literature search, we found limited research examining how SES affects the health conditions of urban and rural residents in China, particularly when considering the mediating role of technological challenges and the moderating effect of urban–rural differences in this relationship.

Accordingly, this study contributes to the current literature by addressing a key challenge: the mastery of new technology, emphasizing technological challenges as a crucial mediation variable in the association between SES and individuals’ health conditions. As technology continues to evolve globally—especially in China—these associations may become increasingly critical [28]. The initial stage (before 2012) was characterized by exploration, with limited focus on medical technology as a core development theme. The second stage (approximately 2012–2015) focused on the informatization of the medical care system, aiming to improve internal efficiency within medical institutions and enhance collaboration across various regions and levels. During this period, most residents continued to access medical care traditionally, minimizing the need for extensive knowledge of medical information technology. The third stage (2016 to present) ushered in the development and implementation of a digitalized medical care system and big data, fundamentally altering residents’ medical treatment patterns. This transformation witnessed the mainstream adoption of practices like online doctor appointments, digital medical insurance payments, and electronic reimbursement vouchers [29]. Despite varying levels of acceptance, most residents were pushed to use these technological services [30], a trend intensified by the COVID-19 pandemic, which increased the role of information technology in providing medical care [6]. These shifts in China’s medical care service system, driven by information technology, highlight an emerging mechanism in the association between SES and residents’ health, making it an increasingly important area of inquiry.

Further, there are considerable differences in rural and urban residents’ access to medical resources in China [31, 32]. Prior research has enhanced our understanding of urban–rural disparities in medical care services [33, 34], but few studies have incorporated a rural–urban variable into research models to investigate the relationship between SES and health conditions, with technological challenges as a mediator. This study contends that the rural–urban factor significantly moderates the effect of technological challenges on health conditions and the mediated effect of SES on health through technological challenges. By examining these hypotheses, our examination of the rural–urban factor’s moderating role provides essential reference data for practical stakeholders striving to improve medical care policy implementation in China.

This study aims to elucidate the relationship between SES and health in the medical sector, contributing new evidence on the diverse impact of technological challenges on rural and urban residents. Moreover, this study proposes a moderated mediation model that sheds light on the conditions under which SES exerts a stronger indirect impact on health through technological challenges.

## Literature review and hypotheses

### SES and health condition

SES can be measured by an individual’s material or nonmaterial resources [35, 36]. People with low SES are characterized by relatively poor knowledge [37–39], low income [21, 40], poor living environment [41, 42], difficulty in obtaining medical care services [43, 44], and experiencing pressure or engaging in short-sighted decision-making [45, 46]. SES is consistently associated with residents’ health conditions [47–51]. For example, Meyer, Castro-Schilo, and Aguilar-Gaxiola [45] proposed that lower SES significantly correlates with weak mental and self-rated health based on an investigation of 44,921 American adults.

The theoretical basis for the association between SES and health conditions is as follows [52, 53]. First, individuals with low SES have limited income, fewer material resources [54], and consume less healthy and nutritious food as compared to their counterparts [50, 55, 56], which may lead to poor health conditions [57]. Second, unhealthy behaviors imply that individuals with low SES tend to engage in unhealthy behaviors [58–60], like smoking, excessive drinking, and lack of exercise [61, 62], exacerbating their health condition [63, 64]. Third, poor decision-making refers to the fact that lower-SES individuals are likely to develop a fast life strategy [65, 66], acting rashly with less long-term self-investment and engaging in short-sighted decision-making behaviors, leading to a passive attitude toward preventive healthcare (such as regular health checks) and low health conditions overall [44, 67, 68]. Fourth, low SES groups face difficulties accessing available medical resources [27, 69, 70], especially in countries where private medical care insurance plays a vital role in medical care cost coverage [71, 72]. Therefore, low SES groups may only have access to relatively low-level medical treatment resources, which could worsen their health conditions [73, 74]. We propose that technological challenges serve as an essential mediator in this association, with a relatively broad impact.

### Technological challenges: a mediator between ses and health condition

The literature provides a theoretical basis for analyzing the mediator between SES and health conditions. SES is closely linked to residents’ ability to access technological information on medical services [75]. For instance, residents with low SES could experience more challenges owing to the lack of electronic equipment and the need to pay a fee for accessing information because their disposable income is relatively low, giving them relatively scarce access to social resources [2]. Compared with high-SES residents, those with low SES have fewer opportunities to learn and practice technological skills, resulting in greater technological challenges when accessing technological information [76]. 

**Fig. 1:**
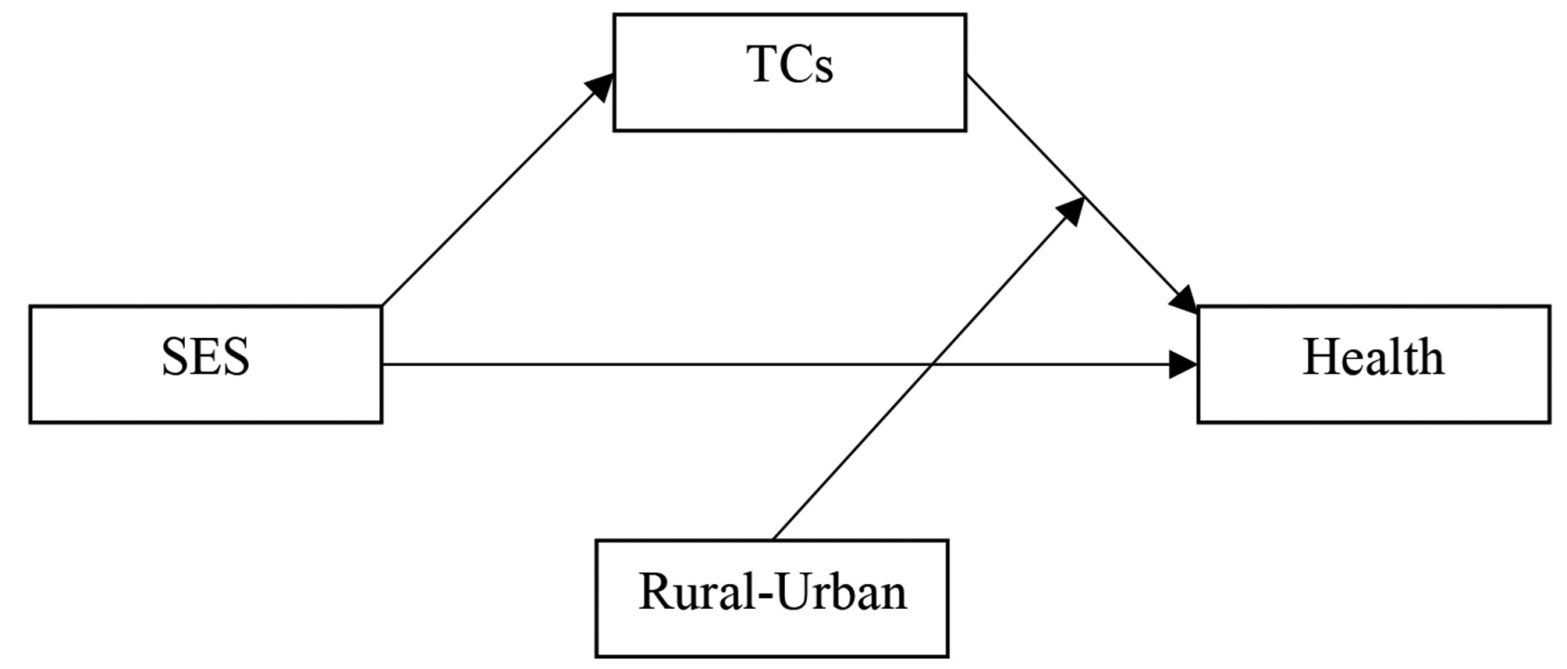
The proposed theoretical model. *Note*. SES: socioeconomic status, TCs: technological challenges

Research has indicated that technological challenges significantly impact residents’ health conditions [7, 13]. First, technological challenges impact residents’ access to healthcare tips and disease prevention information [11]. In China, the government and non-profit organizations have tried to publish healthcare information and care service tips on social media over the last 10 years [77, 78]. However, residents with limited access to modern technology receive less relevant information [79, 80], reducing their chances of receiving comprehensive medical services. Second, medical institutions rely on innovative technology for sharing diagnostic and treatment information [6, 14], requiring patients to make appointments and access screening results online before visiting doctors. Residents with low SES may have difficulties using such technology, potentially exacerbating their health conditions [81]. Third, medical institutions may provide medication instructions and rehabilitation services online, but residents with low SES may struggle to use these services because of their limited knowledge of modern technology [20]. Consequently, they may exhibit passive behavior when engaging in medical services, contributing to poor health conditions [82, 83]. Fourth, an increasing number of hospitals are using information technology to release various diagnoses and test results, and to provide services like medication and rehabilitation [5, 8, 84]. Residents with low SES find it challenging to use these services, leading to poor medical experiences, including face-to-face medical treatment. This leads to our first hypothesis concerning the mediating role of technological challenges, where the mediation effect suggests that the impact of SES on health conditions is at least partially mediated by technological challenges (Fig. 1) [85]. 

#### Hypothesis 1

Technological challenges mediate the association between SES and health conditions.

### Moderating variable: rural–urban

The literature shows that rural residents experience more serious technological challenges than urban residents, partly due to the distribution of medical care resources in rural and urban areas [86–88]. This disparity has been a critical area of research, highlighting significant differences in access to and use of medical resources between rural and urban residents in China [89, 90]. For example, urban residents have greater access to high-quality medical care resources because most high-ranking hospitals are in urban areas [91, 92]. Compared with urban residents, rural residents must spend relatively more time and money to access similar medical resources and benefit from modern information technology [10], such as making appointments or obtaining medical test results online. Rural older adults benefit the least from these changes [93]. Moreover, compared with rural residents, urban residents have more support in using modern information technology related to medical resources, like volunteer assistance, free community-organized information technology training, and home-help services [13]. We thus propose the second hypothesis regarding the moderating role of the rural–urban variable (Fig. 1). The moderating effect suggests that the magnitude and/or direction of the impact of technological challenges on health conditions is affected by whether residents live in urban or rural areas [85]. 

#### Hypothesis 2

The rural–urban factor plays a moderating role in the association between technological challenges and health conditions. Contrastingly, the association between technological challenges and health conditions is stronger among rural residents and relatively weaker among urban residents.

Compared with rural residents, urban residents more frequently use and are more familiar with high-quality medical resources [32, 33, 94]. Urban residents can also more easily acquire and access support from their social network in accessing these efficient medical care resources than rural residents [95, 96]. We propose the third hypothesis based on the above analysis and hypotheses 1 and 2.

#### Hypothesis 3

The mediating effect of SES on health conditions through technological challenges is moderated by the rural–urban factor, with a stronger mediating effect observed among rural residents than among urban residents.

## Methods

### Sample and procedure

This cross-sectional, survey-based research involved 1,932 residents from Anqing and Wuhu in Anhui province, China. These two cities were chosen because they are both third-tier cities, a common city type that better represents the average economic and social development in China [97]. In addition, our research team has an established academic partnership with the Civil Affairs Bureau, Social Security Bureau, and medical institutions in these two cities, facilitating their support and cooperation.

With the support of community workers, university student interviewers who understood the local dialect administered the questionnaires face-to-face. A multi-stage random sampling method was used to recruit respondents. We randomly selected a district (county) in each city, then randomly selected 10 communities from each district and 100 households from each community. If a household declined participation, was unoccupied after three visits, or did not meet eligibility criteria, it was replaced with another household from the remaining list until 100 households were surveyed in each community.

Inclusion criteria were (1) aged ≥ 45 years, because they have relatively high medical care needs and they also have high care responsibilities for family members; [98] (2) living in the community for at least six months in the past year; (3) having basic local medical care insurance, (4) being able to communicate with the interviewers, and (5) willing to participate in the questionnaire and answer the questions after acknowledging the study purpose. A total of 2,000 questionnaires were distributed, 1,958 were returned, 26 incomplete-answered questionnaires were removed, and 1,932 valid questionnaires remained for analysis. Participants were 1,037 men (53.67%) and 891 women (46.12%) (sex information for four participants was missing). Participants’ average age was 64.28 years (*SD*_age_ = 11.30), ranging from 45 to 99 years. The number of urban and rural participants was 792 (40.99%) and 1,140 (59.01%), respectively. Regarding educational level, 47.15% of participants had junior high school or above, 49.07% had primary school or below, and 73 participants did not respond. Around 15.53% of participants had extra commercial medical care insurance in addition to public or social insurance. In the preceding 12 months, 46.33% of participants (or their family members) had received medical reimbursements (i.e., they had used their medical care insurance in the previous year).

### Ethical considerations

This study received prior approval from the Ethics Committee of Anhui Normal University (no. AHNU-ET2022070). Before participating in the interview, participants were informed of the study purpose, nature, and method. They were given the following options: (1) the choice to either participate in or decline the interview; (2) the ability to withdraw from the interview at any time by notifying the interview organizers; (3) assurance that their rights would not be affected by withdrawing from the interview; and (4) assurance that all information gathered during this interview was treated as confidential, and that questionnaires would be securely stored. Under no circumstances were participants’ personal information disclosed to third parties. Written consent was obtained from each participant, who received a compensation of $2.5 upon completing the questionnaire.

## Measures

### SES

We measured SES using a widely used scale developed [99], which has good reliability and validity [100]. This scale contains six items; for example, “I have enough money to buy things I want” and “I don’t need to worry too much about paying my bills.” Responses were rated on a seven-point Likert scale (1 = *strongly disagree*, 7 = *strongly agree*). The Cronbach’s α coefficient of the scale in this study was 0.83. We considered the mean of all items to indicate residents’ SES, with higher scores reflecting higher SES.

### Technological challenges

We developed three items to measure participants’ technological challenges: “I cannot understand the modern technological information systems that I must use to access medical care services”; “I find it difficult to fill various electronic forms to get medical services or provide the necessary information to get reimbursement from my medical care insurance online”; and “I face difficulties in getting help to understand the process of using the technological information system to access medical services.” Responses were rated on a five-point Likert scale (1 = *strongly disagree*, 5 = *strongly agree*). The Cronbach’s α coefficient of this scale was 0.87. The mean value of participants’ scores on the three items was used as the technological challenges index, with higher scores reflecting higher technological challenges.

### Health condition

Participants responded to the following item: [101, 102] “What is your overall perspective of your health condition?” Responses were rated on a seven-point Likert scale (1 = *very bad*, 7 = *very good*). This item was posed to respondents concerning similar research that used similar single-item measurement tools with good reliability and validity [103]. Higher scores indicated better health conditions.

### Control variables

Control variables included participants’ age, sex (1 = male, 0 = female), educational level (1 = junior high school and above, 0 = primary school and below), and marital status (1 = married, 0 = other) in the conceptual model, as the literature states that these demographics are significantly related to health conditions [73, 104]. Moreover, we used a dichotomous variable to measure residents’ living arrangements (1 = living alone, 0 = other).

## Statistical methods

The verified data were imported into SPSS 25.0 (IBM Corp., Armonk, NY, USA) and M-Plus version 8.11 (Muthén & Muthén, Los Angeles, CA, USA) for statistical analysis. First, we conducted descriptive statistical analysis to calculate the mean, standard deviation, and correlation coefficients for the core variables. Second, we tested for common method bias to ensure no severe bias issues in the data. Third, we tested the proposed hypotheses sequentially. We analyzed the direct effect of SES on health conditions and then determined whether technological challenges mediated this effect. Subsequently, we constructed a moderated mediation model to examine whether technological challenges mediated the relationship between SES and health conditions and to assess differences in the impact of technological challenges on health conditions between urban and rural residents [105]. We mean-centered the continuous variables before the analysis [35], and the number of bootstraps was set to 10,000. Unless otherwise specified, control variables (i.e., sex, age, and marital status) were included in the model. Given the low proportion of missing data (far below 5%), we used listwise deletion for the statistical analysis [106]. Significance was set at 0.05.

## Results

### Results of descriptive statistical analysis

The descriptive analysis results, including mean, standard deviation, and correlation coefficient for SES, technological challenges, health conditions, and control variables, are presented in Table 1. Participants’ SES was significantly positively correlated with health conditions and significantly negatively correlated with technological challenges. Technological challenges were significantly negatively correlated with health conditions.

**Table 1:** Means, standard deviations, and correlations for study variables

Variable^a^	*M*	*SD*	1	2	3	4	5	6	7	8	9	10
**1. SES** ^**b**^	3.06	1.18										
**2. TCs** ^**c**^	3.60	1.02	− 0.23 (< .001^K^)									
**3. Health** ^**d**^	5.03	1.39	0.15 (< .001^K^)	− 0.15 (< .001^K^)								
**4. RU** ^**e**^	0.41	0.49	0.26 (< .001^K^)	− 0.17 (< .001^K^)	− 0.02 (.48^K^)							
**5. Sex**	0.54	0.49	− 0.01 (.93^K^)	− 0.04 (.12^K^)	0.07 (.004^K^)	0.06 (.006^K^)						
**6. Age**	64.28	11.30	− 0.03 (.15^K^)	− 0.25 (< .001^K^)	− 0.15 (< .001^K^)	0.06 (.01^K^)	0.06 (.01^K^)					
**7. Edu** ^**f**^	0.49	0.50	0.28 (< .001^K^)	− 0.28 (< .001^K^)	0.07 (.002^K^)	0.41 (< .001^K^)	0.17 (< .001^K^)	− 0.28 (< .001^K^)				
**8. Marr** ^**g**^	0.89	0.31	0.05 (.02^K^)	− 0.13 (< .001^K^)	0.06 (.01^K^)	0.04 (.054^K^)	0.03 (.28^K^)	− 0.22 (< .001^K^)	0.18 (< .001^K^)			
**9. Alone** ^**h**^	0.14	0.35	− 0.06 (.01^K^)	0.12 (< .001^K^)	− 0.08 (.001^K^)	− 0.05 (.02^K^)	0.00 (.99^K^)	0.20 (< .001^K^)	− 0.19 (< .001^K^)	− 0.53 (< .001^K^)		
**10. CoSu** ^**i**^	0.16	0.36	0.08 (< .001^K^)	− 0.09 (< .001^K^)	0.02 (.50^K^)	0.06 (.009^K^)	0.03 (.20^K^)	0.22 (< .001^K^)	0.16 (< .001^K^)	0.05 (.03^K^)	− 0.02 (.44^K^)	
**11. Utilize** ^**j**^	0.46	0.49	0.05 (.02^K^)	− 0.01 (.78^K^)	0.13 (< .001^K^)	− 0.04 (.07^K^)	0.05 (.03^K^)	0.03 (.19^K^)	0.03 (.20^K^)	0.05 (.04^K^)	− 0.04 (.10^K^)	0.10 (< .001^K^)

^a^*N* = 1,832, ^b^SES: socioeconomic status, ^c^TCs: technological challenges, ^d^Health: self-rated health condition, ^e^RU: rural–urban, ^f^Edu: educational level,^g^Marr: marital status, ^h^Alone: living alone, ^i^CoSu: commercial insurance, ^j^Utilize: healthcare policy utilization in the past 12 months, ^K^PV: *p*-value

### Common method bias test

To assess common method bias, we used Harman’s single factor test [107, 108]. We conducted an exploratory factor analysis for SES, technological challenges, health conditions, and the rural–urban variable. The results indicated that a single factor only explained 22.66% of the total variance, much lower than the recommended standard [107]. This suggests that common method bias was not a concern.

### Results for hypothesis 1

We first examined the direct effect of SES on health conditions. SES had a significant positive effect on health conditions (*B* = 0.17, *p* < .001), which aligns with the results of several SES studies [50, 57, 64]. The 95% confidence interval (CI) from 10,000 bootstrapped samples, [0.11, 0.23] (excluding zero), further confirmed this significant positive effect.

SES had a significant negative impact on technological challenges, technological challenges had a significant negative impact on health conditions, and the indirect effect of SES on health conditions through technological challenges was significant (Table 2). Since the 95% CI did not include zero, technological challenges were found to mediate the association between SES and health conditions. Moreover, the direct effect of SES on health remained significant after adding technological challenges as a mediator, indicating partial mediation. The indirect effect of SES on health conditions through technological challenges accounted for approximately 10.0% of the total effect of this relationship. Thus, Hypothesis 1 was supported.

**Table 2:** Results of the simple mediation model

Variables ^a^	*B*	*SE*	*p*	Bootstrapped 95% CI^b^	
				*LL* ^c^	*UL* ^d^
**Mediator variable model: TCs** ^e^					
SES^f^	-0.15	0.02	< 0.001	-0.19	-0.10
Sex	-0.01	0.02	0.84	-0.10	0.08
Age	0.02	0.002	< 0.001	0.01	0.02
Edu^g^	-0.33	0.05	< 0.001	-0.42	-0.23
Marr^h^	-0.14	0.09	0.10	-0.31	0.03
Alone^i^	0.06	0.08	0.42	-0.08	0.22
CoSu^j^	-0.04	0.06	0.50	-0.16	0.08
Utilize^k^	0.03	0.05	0.47	-0.06	0.12
**Outcome variable model: Health** ^l^					
SES	0.15	0.03	< 0.001	0.10	0.21
TCs	-0.12	0.03	0.001	-0.18	-0.05
Sex	0.21	0.06	0.001	0.09	0.33
Age	-0.02	0.003	< 0.001	-0.02	-0.01
Edu	-0.10	0.07	0.15	-0.23	0.03
Marr	-0.02	0.12	0.90	-0.26	0.23
Alone	-0.23	0.11	0.04	-0.45	-0.01
CoSu	-0.06	0.08	0.48	-0.22	0.11
Utilize	-0.38	0.06	< 0.001	-0.50	-0.26
SES à TCs à Health^m^	0.02	0.01	0.002	0.01	0.03

^a^*N* = 1,821, ^b^CI: confidence interval, ^c^LL: lower limit, ^d^UL: upper limit, ^e^TCs: technological challenges, ^f^SES: socioeconomic status, ^g^Edu: educational level, ^h^Marr: marital status, ^i^Alone: living alone, ^j^CoSu: commercial insurance, ^k^Utilize: healthcare policy utilization in the past 12 months, ^l^Health: self-rated health condition,^m^SES à TCs à Health: SES effect on health condition through TCs

### Results for hypothesis 2

Technological challenges significantly negatively influenced health conditions after adding the rural–urban variable and the cross-product term between technological challenges and the rural–urban variable (*B* = -0.21, *p* < .001, 95% CI [-0.29, -0.12]). The interaction term also significantly affected health conditions (*B* = 0.19, *p* = .004, 95% CI [0.06, 0.32]; Table 3).

**Table 3:** Results of the moderated mediation model

Variables ^a^	*B*	*SE*	*p*	Bootstrapped 95% CI^b^	
				*LL* ^c^	*UL* ^d^
**Mediator variable model: TCs** ^e^					
SES^f^	-0.15	0.02	< 0.001	-0.19	-0.11
Sex	-0.01	0.05	0.85	-0.10	0.08
Age	0.02	0.01	< 0.001	0.01	0.02
Edu^g^	-0.33	0.05	< 0.001	-0.42	-0.23
Marr^h^	-0.14	0.09	0.10	-0.31	0.03
Alone^i^	0.06	0.08	0.40	-0.09	0.21
CoSu^j^	-0.04	0.06	0.49	-0.15	0.07
Utilize^k^	0.03	0.05	0.47	-0.06	0.12
**Outcome variable model: Health** ^l^					
SES	0.17	0.03	< 0.001	0.11	0.23
TCs	-0.21	0.05	< 0.001	-0.29	− 0.012
RU	-0.15	0.07	0.03	-0.29	-0.01
TCs*RU^m^	0.19	0.07	0.004	0.06	0.32
Sex	0.19	0.06	0.003	0.06	0.32
Age	-0.02	0.01	< 0.001	-0.02	-0.01
Edu	-0.06	0.07	0.45	-0.20	0.09
Marr	0.01	0.13	0.92	-0.24	0.26
Alone	-0.25	0.11	0.03	-0.48	-0.04
CoSu	-0.05	0.08	0.53	-0.21	0.11
Utilize	-0.37	0.06	< 0.001	-0.49	-0.25

^a^*N* = 1,794, ^b^CI: confidence interval, ^c^LL: lower limit, ^d^UL: upper limit, ^e^TCs: technological challenges, ^f^SES: socioeconomic status, ^g^Edu: educational level, ^h^Marr: marital status, ^i^Alone: living alone, ^j^CoSu: commercial insurance, ^k^Utilize: healthcare policy utilization in the past 12 months, ^l^Health: self-rated health condition,^m^RU: rural–urban

**Fig. 2:**
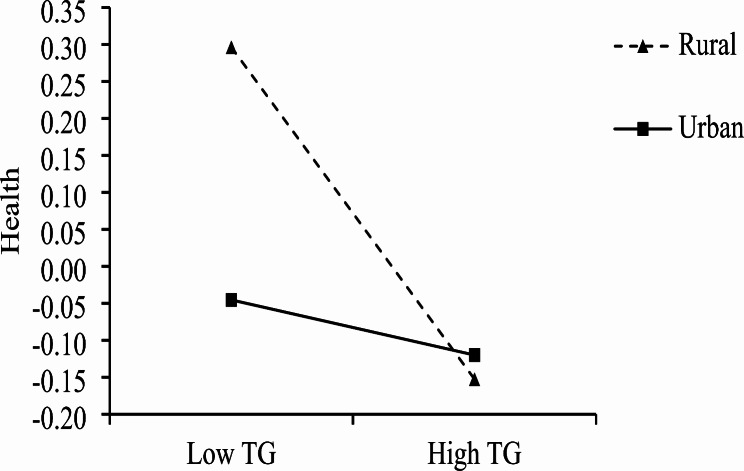
Interaction between technological challenges and location on health. *Note*. TCs: technological challenges

We conducted a simple slope analysis to analyze the moderating effect of the rural–urban variable on the effect of technological challenges on health conditions [109] (Fig. 2). Urban residents’ (rural–urban = 1) technological challenges had no significant effect on health condition (*B* = -0.02, *p* = .68, 95% CI [-0.11, 0.08]), whereas rural residents’ (rural–urban = 0) technological challenges had a significant negative effect on health conditions (*B* = -0.21, *p* < .001, 95% CI [-0.29, -0.12]).

We also constructed an index to represent the difference in the regression coefficients of technological challenges on health conditions between urban and rural residents. The 95% CI of this index, calculated using 10,000 bootstrap samples, was [0.06, 0.32], excluding zero, indicating a significant difference between the two regression coefficients. Thus, Hypothesis 2 was partially supported (Table 3), with technological challenges having a greater impact on rural residents’ health conditions than on those of urban residents.

### Results for hypothesis 3

Based on the recommendations of statisticians [105, 110], we examined whether the rural–urban variable moderated the mediated effect of SES on health conditions through technological challenges. The mediated effect of SES on health conditions through technological challenges for urban residents was not significant (*B* = 0.003, *p* = .41, 95% CI [-0.01, 0.02]), but it was significant for rural residents (*B* = 0.03, *p* < .001, 95% CI [0.02, 0.05]). We also constructed an index to represent the difference between the intermediary effect of urban and rural residents. Using 10,000 bootstrapped samples, the 95% CI of the index was calculated as [-0.05, -0.01], excluding zero. Consequently, Hypothesis 3 was supported.

### Outcomes without control variables

We examined whether the mediating effect of SES on health conditions through technological challenges remained significant in a simple mediation model without the control variables (age, sex, marital status, and other control variables). In the direct model, the effect of SES on health conditions remained significant without control variables (*B* = 0.18, *p* < .001, 95% CI [0.12, 0.23]). In the mediation model, the effect of SES on health conditions through technological challenges remained significant without control variables (*B* = 0.03, *p* < .001, 95% CI [0.02, 0.05]). In the moderated mediation model without control variables, the cross-product term between technological challenges and the rural–urban variable was significant (*B* = 0.19, *p* = .003, 95% CI [0.07, 0.32]). The mediating effect of SES on health conditions through technological challenges was not significant for urban residents (*B* = 0.01, *p* = .16, 95% CI [-0.01, 0.03]), but was significant for rural residents (B = 0.05, *p* < .001, 95% CI [0.03, 0.07]). These results indicate that the rural–urban variable moderated the effect of SES on health conditions through technological challenges. Consistent with results from models including the control variables, these findings demonstrate the strong robustness of the proposed model.

## Discussion

Given the widespread implementation of information technology in medical care services in China, which has transformed the delivery and utilization of medical services, we argue that SES affects health conditions through the mediating role of technological challenges. We proposed that technological challenges mediate the effect of SES on Chinese urban and rural residents’ health conditions. By examining this hypothesis, this study provides novel insights into the effect of SES on health and contributes new evidence on how technological challenges differently affect on rural and urban residents. In contrast to prior studies that emphasized the importance of further examining how much SES impacts people’s health conditions through technological challenges [21], our findings clarify this understanding by demonstrating that individuals with lower (vs. higher) SES face greater technological challenges, resulting in poorer health conditions. Moreover, the rural–urban factor moderates the mediated effect of SES on health conditions, with a stronger effect among rural residents. Rural individuals with low SES have poorer health conditions because of technological challenges.

First, our results reveal a new mechanism: technological challenge the extent to which residents with low SES experience changes in health conditions when accessing and using medical services and resources. While the extensive use of advanced information technologies in medical care services enhances delivery efficiency, it appears to widen the gap between urban and rural residents, as rural residents are often less equipped to use modern information technology systems. These findings underscore the importance of considering whether and how modern technology can be integrated into traditional medical service delivery methods for certain groups based on their medical care needs. These findings have implications for health policymakers and highlight the need to bridge the gap between medical resource delivery methods and health improvements among older adults.

Second, our findings expand on the topic by showcasing that rural residents experience strong technological challenges regarding medical care service use, which worsens their health conditions and enhances our understanding of urban–rural differences in medical resource use and health outcomes among Chinese residents. Specifically, limited access to medical resources and services hinders the efficiency of medical care policy implementation in rural areas [89, 92]. Rural residents’ SES can significantly weaken their health condition through technological challenges, advancing the scientific evidence on how rural residents’ technological challenges affect their health conditions.

Third, we suggest that practical stakeholders consider the role of technological challenges when analyzing the SES–health condition relationship, as this could significantly support information technology system development, simplification, and ease of use within China’s medical care services, reducing learning costs for residents. Providing residents with low SES—particularly in rural areas—greater access to basic electronic equipment and organizing local community training on using medical information technology could help reduce technological challenges and improve health conditions. Moreover, to mitigate the negative impact of low SES on health conditions through technological challenges, the information literacy of residents with low SES should be improved [76]. Additionally, retaining some traditional medical services, especially in rural areas, could help them residents better adapt to modern technologies used in medical services and decrease their risk of worsening health conditions.

Despite these strengths, this study has some limitations. First, its cross-sectional design limits causal inference, and we only included adults aged 45 years or older in the Anhui province of China. Future research could consider longitudinal designs, such as cross-lagged designs, and include diverse populations from different regions and cultural backgrounds worldwide to explore the mediating role of technological challenges in the SES–health condition relationship. Second, this study used simplified measurement items to avoid participant fatigue if they, especially since many participants were older. We also assessed participants’ health conditions and SES using subjective scales, which may limit the findings.

Notably, previous studies demonstrated the high reliability and validity of short-form scales and objective SES measurement tools. Future research could elucidate these findings by using more comprehensive measurement tools and incorporating objective SES measures. Third, while this research highlighted technological challenges as an essential mechanism in the informatization of medical care service delivery, scholars should expand the sampling range and conduct comparative analyses to explore different mechanisms underlying the relationship between SES and health conditions, while retaining the current research focus. Fourth, while this study controlled for important variables (e.g., age, educational level, living arrangement, and medical care resource use in the last 12 months), it did not consider factors like health literacy, which should be incorporated as a control variable in future models to expand our knowledge of the associations examined in this study. We intend to include this variable in our forthcoming research. Fifth, this study does not investigate whether mitigating technological challenges can alleviate the effect of SES on residents’ health conditions. To explore this, a randomized controlled research design could be employed to assess to what extent targeted interventions can alleviate technological challenges among residents with low SES and whether these interventions can lessen the negative impact of SES on health conditions.

## Conclusion

This study investigates the impact of SES on health conditions through technological challenges and identifies the differences between rural and urban residents in China. Since low SES can lead to adverse effects health outcomes, understanding how technological challenges exacerbate these health conditions is important for developing effective medical care policies. We found that the SES of older adult residents had a significant negative impact on their health, partly owing to technological challenges, with distinct rural–urban differences in this relationship.

These insights provide key theoretical contributions by focusing on the effect of SES within a context relevant to medical care policy formulation and implementation. Given the ever-increasing costs associated with integrating modern technologies into medical care service delivery, the consequences of inefficient implementation and underutilization are relevant. This study provides evidence and insights at the intersection of policy formulation, innovative technology, and public perceptions, addressing potential shortcomings and risks involved in public health policy.

## Data Availability

The datasets generated and analyzed in this study are not publicly available. Anonymized data can be obtained from two sources: from the local government, which provided financial support for this study; and from the corresponding author upon reasonable request and with permission from the School of Educational Science, Anhui Normal University, China.

## Abbreviations

CI: Confidence interval
SES: Socioeconomic status
